# Pemphigus and Bullous Pemphigoid Following COVID-19 Vaccination: A Systematic Review

**DOI:** 10.3390/v16121896

**Published:** 2024-12-09

**Authors:** Fabrizio Martora, Teresa Battista, Luca Potestio, Maddalena Napolitano, Cataldo Patruno, Matteo Megna, Michela D’Agostino

**Affiliations:** 1 Section of Dermatology, Department of Clinical Medicine and Surgery, University of Naples Federico II, Via Pansini 5, 80131 Napoli, Italy; teresabattista12@gmail.com (T.B.); potestioluca@gmail.com (L.P.); maddy.napolitano@gmail.com (M.N.); mat24@libero.it (M.M.); dagos.michela@gmail.com (M.D.); 2 Department of Health Sciences, University Magna Graecia of Catanzaro, 88100 Catanzaro, Italy; cataldopatruno@libero.it

**Keywords:** COVID-19 vaccine, immune-mediated inflammatory disease, pemphigus vulgaris, pemphigus foliaceus, bullous pemphigoid, safety, skin, adverse events

## Abstract

The COVID-19 pandemic has encouraged the rapid development and licensing of vaccines against SARS-CoV-2. Currently, numerous vaccines are available on a global scale and are based on different mechanisms of action, including mRNA technology, viral vectors, inactive viruses, and subunit particles. Mass vaccination conducted worldwide has highlighted the potential development of side effects, including ones with skin involvement. This review synthesizes data from 62 manuscripts, reporting a total of 142 cases of autoimmune blistering skin diseases (AIBDs) following COVID-19 vaccination, comprising 59 cases of pemphigus and 83 cases of bullous pemphigoid. Among the 83 bullous pemphigoid cases, 78 were BP, with additional cases including 2 oral mucous membrane pemphigoid, 1 pemphigoid gestationis, 1 anti-p200 BP, and 1 dyshidrosiform BP. The mean age of affected individuals was 72 ± 12.7 years, with an average symptom onset of 11 ± 10.8 days post-vaccination. Notably, 59% of cases followed vaccination with BNT162b2 (Pfizer-BioNTech), 51.8% were new diagnoses, and 45.8% occurred after the second dose. The purpose of our review is to analyze the cases of pemphigus and bullous pemphigoid associated with COVID-19 vaccination and to investigate the pathogenetic mechanisms underlying the new development or flare-up of these diseases in association with vaccination. Our results show that the association between COVID-19 vaccines and AIBDs is a possible event.

## 1. Introduction

Mucocutaneous adverse events have emerged as some of the most frequently reported reactions associated with COVID-19 vaccination, drawing considerable attention from the medical community. These reactions encompass a broad spectrum of clinical presentations, which can be classified into various categories depending on the underlying pathogenetic mechanisms. These mechanisms include hypersensitivity reactions, notably types I and IV, which involve immediate and delayed immune responses, respectively. Additionally, local site reactions at the injection site, autoimmune-mediated reactions, functional angiopathies, and the reactivation of latent viral conditions have been identified as potential contributors to these adverse skin manifestations following the administration of COVID-19 vaccines [1,2].

Within the broader scope of autoimmune diseases, there has been a notable increase in reports of new onset or recurrence of autoimmune bullous diseases (AIBDs) following COVID-19 vaccination. These diseases, characterized by the formation of blisters on the skin and mucous membranes, are particularly concerning due to their chronic nature and the significant impact they have on patients’ quality of life [3]. Among the various types of AIBD, pemphigus and bullous pemphigoid (BP) are the most prominent, each with distinct pathophysiological features. Pemphigus is characterized by the presence of autoantibodies directed against desmosomes, the structures that facilitate cell-to-cell adhesion within the epidermis, leading to intraepidermal blistering. In contrast, BP involves autoantibodies targeting hemidesmosomes, which anchor the epidermis to the underlying basement membrane, resulting in subepidermal blistering.

Given the increasing number of reported cases, there is a pressing need to understand the potential relationship between COVID-19 vaccination and the onset or exacerbation of these diseases. The aim of our study was to conduct a comprehensive review of the recent literature focusing on cases of new diagnoses and flare-ups of pemphigus and BP following COVID-19 vaccination. By examining these cases, we seek to elucidate any patterns or trends that may contribute to a better understanding of the potential risks associated with COVID-19 vaccines in relation to these serious autoimmune conditions [4].

## 2. Methods

Comprehensive literature research was conducted on MEDLINE/PubMed, Web of Science, embase, scopus, and Google Scholar databases until May 2024. The following keywords were used to research data: “bullous pemphigoid”, “pemphigus”, “pemphigus vulgaris (PV”), “pemphigus foliaceus (PF)”, “autoimmune bullous disease”, “immune-mediated inflammatory disease”, “COVID-19”, “vaccination”, “vaccine”, “skin manifestations”, “adverse events”, “Pfizer/BioNTech”, “BNT162b2”, “Moderna”, “mRNA-1273”, “AstraZeneca”, “AZD1222”, “Covishield”, “Johnson & Johnson”, “Ad26.COV2.S”, “BBIBP-CorV”, “Sinopharm”, “CoronaVac”, “mRNA”, “viral-vector”, “cutaneous”, “side effect”. Data from the selected case reports and case series were analyzed, and a total of 62 articles were found to be relevant. The references of the selected manuscripts were evaluated to include articles that could have been missed. Our analysis included EMA-approved vaccines, and the main vaccines spread globally.

**Figure 1:**
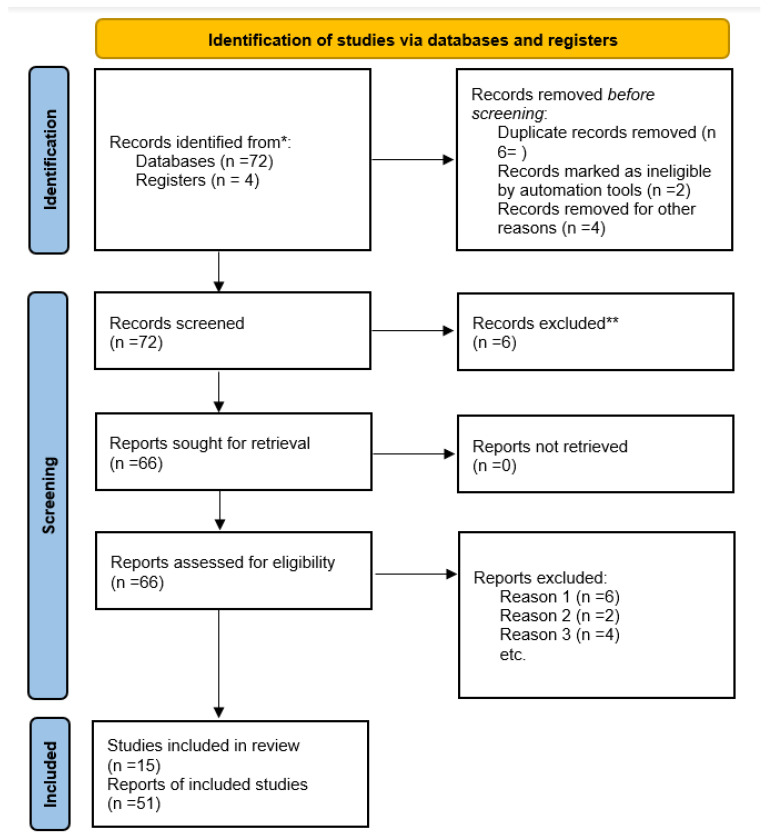
PRISMA 2020 flow diagram for new systematic reviews that include searches of databases and registers only. * Consider, if feasible to do so, reporting the number of records identified from each database or register searched (rather than the total number across all databases/registers). ** If automation tools were used, indicate how many records were excluded by a human and how many were excluded by automation tools. From: Page MJ, McKenzie JE, Bossuyt PM, Boutron I, Hoffmann TC, Mulrow CD, et al. The PRISMA 2020 statement: an updated guideline for reporting systematic reviews. BMJ 2021;372:n71. doi: 10.1136/bmj.n71 [5]. For more information, visit: http://www.prisma-statement.org/.

The selection and extraction of relevant data followed the PRISMA (Preferred Reporting Items for Systematic Reviews and Meta-Analyses) guidelines (Figure 1).

### 2.1. Study Selection

In the first step, two researchers (M.D. and F.M.) reviewed the retrieved articles and removed the duplicates. In other steps, the researchers screened the title and abstract of the records, and the ineligible studies were removed. Then, the authors surveyed the full text of the remaining studies based on inclusion and exclusion criteria, and the eligible studies were identified.

We decided to exclude certain articles based on the following criteria: articles that were not original research, such as reviews or editorials; articles for which the full text was unavailable; papers that were only available as abstracts, including conference abstracts; clinical trials that were still ongoing and had not yet published results; articles written in languages other than English.

### 2.2. Risk of Bias Selection

The quality of enrolled studies was appraised by two investigators (F.M. and T.B) using the revised Cochrane risk-of-bias tool for letter to editor, case report, case series, and reviews. Through evaluating the methodology, the overall risk of bias was judged as low, high, or of some concern. If differing opinions existed, the third author (M.M.) was consulted for arbitration.

## 3. Result

Data resulting from the 62 reviewed manuscripts are synthesized in Table 1. A total of 59 cases of pemphigus and 83 of BP following COVID-19 vaccination were collected, amounting to a total of 142 cases of autoimmune blistering skin diseases (AIBDs). Data on the vaccination dose and the time to onset the symptoms refer to the last dose of vaccine administered before the onset of clinical manifestations.

Data on registered cases of BP are reported in Table 2. A total of 83 cases of pemphigoid were registered, including 78 BP cases, 2 oral mucous membrane pemphigoid cases, 1 case of pemphigoid gestationis, 1 case of anti-p200 BP, and 1 case of dyshidrosiform BP. Among these, 46 cases involved the male population (55.42%). The mean age was 72 ± 12.7, while the average time of onset of symptoms was 11 ± 10.8 days. Most cases occurred following vaccination with BNT162b2 (Pfizer-BioNTech) [49, (59%)]. The new diagnoses were 43 (51.8%), and pemphigoid cases occurred mainly after the administration of the second dose [38 (45.8%)].

Data on registered cases of pemphigus are reported in Table 3. A total of 59 cases of pemphigus were registered, including 33 cases of PV, 19 cases of PF, 1 case of pemphigus vegetans, 1 case of pemphigus erythematosus, 1 case of oral pemphigus, 1 case of IgA pemphigus, and 3 cases of an unspecified subtype of pemphigus. The mean age was 55 ± 16.6, while the average time of onset of symptoms was 14 ± 11.7 days from the last vaccination. The female population was involved in a higher percentage [34, (57.6%)]. Most cases developed as a result of vaccinations with BNT162b2 (Pfizer-BioNTech) [29, (49.2%)] and mainly after the administration of the second dose [24, (40.7%)].

## 4. Discussion

Bullous pemphigoid (BP) is an autoimmune blistering disease involving autoantibodies against BP180 and BP230 proteins in the basement membrane. Triggers include UV radiation, trauma, drugs, malignancies, and vaccinations. Cases have been associated with vaccines for influenza, tetanus, diphtheria, polio, pertussis, meningococcal disease, pneumococcal disease, hepatitis B, and rabies. Pemphigus, another autoimmune disorder affecting the skin and mucous membranes, can also be triggered by infections, radiation, drugs, vaccines, pregnancy, or stress, particularly in genetically predisposed individuals. Vaccines such as those for influenza, hepatitis B, rabies, and tetanus have been reported to trigger or exacerbate the disease [65,66,67,68,69,70,71,72].

It is well-established that vaccinations and infections can exacerbate autoimmune diseases, including after COVID-19 vaccines [73,74,75,76,77,78,79,80,81,82,83,84,85]. Numerous new-onset autoimmune diseases, such as systemic lupus erythematosus, Guillain-Barré syndrome, autoimmune liver diseases, and rheumatoid arthritis, have been reported post-COVID-19 vaccination. Among autoimmune skin conditions, cases of vitiligo, alopecia areata, lupus, pityriasis rosea, herpes zoster, leukocytoclastic vasculitis, and autoimmune blistering diseases (AIBD) have also been noted in a systematic review by Ghanaatpisheh et al. [86].

BP and pemphigus can be triggered by environmental factors stimulating autoimmunity in susceptible individuals. COVID-19 vaccines may contribute through mechanisms such as epitope spreading, molecular mimicry, and bystander activation. A systematic review suggests that molecular mimicry between the spike protein and basement membrane components, like BP230 and BP180, could lead to autoantibody production. Vaccination also strongly activates T-helper lymphocytes, cytokine production, autoantibody generation, and complement pathway activation, causing basement membrane damage.

Pemphigus cases post-vaccination are similarly linked to mechanisms like molecular mimicry and bystander activation, with a potential role for CD8+ lymphocytes and the Fas/FasL pathway [33]. Our findings show an increased number of BP and pemphigus cases following mRNA vaccines, particularly BNT162b2, consistent with other studies [78,79,80,81,82,83,84,85,86,87].

There are cases in the literature to report regarding the occurrence or exacerbation of pemphigus following other vaccines. Regarding PV, Cozzani et al. reported a case of pemphigus vulgaris (PV) that developed following tetanus and diphtheria vaccination in an 11-year-old girl. Additionally, Berkun et al. and Mignogna et al. documented two separate instances of PV occurring after vaccination, with one emerging 3 months post-hepatitis B vaccination and the other 1 month after receiving the influenza vaccine [88,89,90].

Other cases are reported as exacerbation of pre-existing PV after influenza vaccination [91].

A different discussion exists for BP because there are no definite and reported data of association in the literature but only reports mostly in pediatric age following the combination of various vaccines. Therefore, with respect to PV, it is not possible to correlate it with one type of vaccine [92,93,94,95].

The mechanisms that cause relapse after vaccination remain uncertain. Two main hypotheses have been proposed: (i) an overactive immune response in individuals with a genetic predisposition, possibly leading to the development of anti-desmoglein antibodies, and (ii) cross-reactivity between vaccine antigens and pemphigus antigens. While there is no definitive evidence supporting these theories in pemphigus or other immune-mediated blistering conditions, such mechanisms have been suggested in the vaccine-induced worsening of other autoimmune diseases [96].

Influenza vaccination is generally recommended for patients on immunosuppressive therapy; however, it may trigger a flare-up of the underlying disease in individuals receiving treatment for autoimmune disorders [96,97,98,99,100].

As reported for COVID-19 vaccination, we can certainly say that although the association may also be present with other vaccines, the benefit of the vaccine itself far outweighs the risk of both occurrence and exacerbation.

## 5. Conclusions

COVID-19 vaccines were developed to overcome the pandemic period, and the mass administration of such vaccines has led to adverse reactions not often detected in clinical trials. The association between COVID-19 vaccines and AIBD is a possible event. However, the exact correlation between the development of bullous diseases and vaccine administration requires clarification and needs further investigation. The identification of a potential causal relationship between vaccination and the occurrence or recurrence of these diseases is important for assessing the risk and counseling patients.

## Figures and Tables

**Table 1:** Pemphigus and BP following COVID-19 vaccination. BP: bullous pemphigoid; PV: pemphigus vulgaris (PV); PF: pemphigus foliaceus.

Authors	Number of Cases	Clinical Manifestations	Patient (Age/Sex)	COVID-19 Vaccination, Vaccination Dose	Days to Onset the Symptoms	New Onset or Recurrence
Shakoei et al. [3]	4	PV	28, F	Sinopharm First dose	14	Recurrence
		PV	30, F	Sinopharm First dose	16	New onset
		BP	85, F	Sinopharm First dose	20	New onset
		BP	91, M	Sinopharm First dose	19	New onset
Rasner et al. [4]	3	BP	88, M	Pfizer/BioNTech Second dose	1	Recurrence
		BP	69, M	Moderna Second dose	14	Recurrence
		PF	50, F	Pfizer/BioNTech First dose	7	Recurrence
Baffa et al. [6]	1	BP	91, F	Pfizer/BioNTech Second dose	10	New onset
Calabria et al. [7]	1	Oral mucous membrane pemphigoid	72, F	Pfizer/BioNTech Third dose	9	New onset
Yamamoto et al. [8]	1	BP	72, M	Pfizer/BioNTech Second dose	Unspecified	New onset
Sun et al. [9]	1	BP	79, F	Pfizer/BioNTech Second dose	3	New onset
Dell’Antonia et al. [10]	1	BP	83, M	Pfizer/BioNTech First dose	7	New onset
Martora et al. [11]	4	BP	60, M	Pfizer/BioNTech second dose	7	Recurrence
			74, M	Pfizer/BioNTech second dose	5	Recurrence
			78, M	Pfizer/BioNTech second dose	8	Recurrence
			80, F	Moderna First dose	6	Recurrence
Corrá et al. [12]	5	PV	61, F	Pfizer/BioNTech Third dose	3	New onset
		PF	80. M	Pfizer/BioNTech Third dose	17	New onset
		PF	66, F	Pfizer/BioNTech Second dose	28	New onset
		PV	73, F	Pfizer/BioNTech Third dose	28	New onset
		PV	63, F	AstraZeneca First dose	28	New onset
Cowan et al. [13]	10	BP: 7	Medium age: 75.1 Sex: F:2 M: 5	Pfizer/BioNTech: 3 Unspecified dose:3 AstraZeneca:4 Second dose:4	Medium time: 55.4	New onset: 4 Recurrence: 3
		Pemphigus: 3	Medium age:51.3 Sex: F: 2 M: 1	Pfizer/BioNTech:3 Unspecified dose:3	Medium time: 37.6	New onset: 1 Recurrence: 2
Diab et al. [14]	4	BP	70, F	Sinopharm First dose	20	New onset
		PF	30, F	Sinopharm Second dose	14	New onset
		BP	77, F	Sinopharm Second dose	30	New onset
		PV	45, M	BIv1-CoViran Second dose	20	New onset
Maronese et al. [15]	21	BP	F: 9 M: 12, Medium age: 82	Pfizer/BioNTech: 17 AstraZeneca: 2 Moderna: 2 First dose: 8 Second dose: 13	Medium time: Unspecified	Unspecified
Rouatbi et al. [16]	2	PF	70, M	Pfizer/BioNtech First dose	7	New onset
			48, M	AstraZeneca First dose	5	New onset
Young et al. [17]	1	BP	68, M	Pfizer BioNTech First dose	3	New onset
Bailly-Caillé [18]	1	anti-p200 BP	74, M	Moderna Second dose	2	New onset
Knechtl et al. [19]	1	PV	89, M	Pfizer BioNTech second dose	30	New onset
Pauluzzi et al. [20]	1	BP	46, M	Pfizer/BioNTech first dose	15	New onset
Avallone et al. [21]	1	PV	46, M	Pfizer/BioNTech First dose	5	Recurrence
Damiani et al. [22]	5	BP	63, F	Moderna First dose	3	Recurrence
		PV	40, M	Moderna first dose	3	Recurrence
		BP	84, M	Moderna first dose	14	Recurrence
		BP	82, F	Pfizer/BioNTech First dose	3	Recurrence
		PV	80, M	Pfizer/BioNTech First dose	3	Recurrence
Hung et al. [23]	1	BP	39, M	Moderna first dose	30	New onset
Ligrone et al. [24]	1	PV	56, F	Moderna Third dose	5	Recurrence
Lua et al. [25]	1	PF	83, M	Pfizer/BioNTech Second dose	2	New onset
Hali et al. [26]	5	BP	51, M	AstraZeneca Second dose	7	New onset
		BP	54, F	AstraZeneca First dose	3	New onset
		BP	68, M	AstraZeneca Second dose	7	New onset
		PF	50, F	Pfizer/BioNTech Second dose	15	New onset
		PV	58, F	Pfizer/BioNTech First dose	30	New onset
Hinterseher et al. [27]	4	PV: 1	36, F	Pfizer/BioNTech Third dose	7	Recurrence
		BP: 3	66, M	Pfizer/BioNTech First dose	21	New onset
			67, M	AstraZeneca First dose	7	Recurrence
			61, F	Pfizer/BioNTech Third dose	Unspecified	Recurrence
Schmidt et al. [28]	1	BP	84, F	Moderna first dose	Unspecified	New onset
Pérez-López et al. [29]	1	BP	78, F	Pfizer/BioNTech First dose	3	New onset
Alshammari et al. [30]	1	BP	78, M	Pfizer/BioNTech Second dose	1	New onset
Desai et al. [31]	1	BP	73, F	Moderna Second dose	1	New onset
Agharbi et al. [32]	1	BP	77, M	AstraZeneca First dose	1	New onset
Aryanian et al. [33]	1	PV	43, M	AstraZeneca Second dose	2	New onset
Hatami et al. [34]	2	PV	34, M	AstraZeneca Unspecified dose	Unspecified	New onset
		PV	61, M	AstraZeneca Unspecified dose	7	Recurrence
Pham et al. [35]	2	PF	53, F	AstraZeneca Fourth dose	21	New onset
		PF	30, F	Moderna Second dose	60	New onset
Bostan et al. [36]	1	BP	67, M	Unspecified First dose	35	New onset
Maronese et al. [37]	3	BP	67, M	Pfizer/BioNTech First dose	Unspecified	New onset
		BP	84, F	Pfizer/BioNTech First dose	Unspecified	New onset
		BP	86, M	Pfizer/BioNTech First dose	Unspecified	New onset
Solimani et al. [38]	1	PV	40, F	Pfizer/BioNTech First dose	5	New onset
Singh et al. [39]	1	PV	40, M	Covishield Second dose	7	New onset
Koutlas et al. [40]	1	PV	60, M	Moderna Second dose	7	New onset
Thongprasom et al. [41]	1	Oral pemphigus	38, F	AstraZeneca First dose	7	New onset
Shanshal [42]	1	Dyshidrosiform BP	90, F	Pfizer/BioNTech First dose	7	New onset
Corrá et al. [12]	5	PV	61, F	Pfizer/BioNTech Third dose	3	New onset
		PF	80, M	Pfizer/BioNTech Third dose	17	New onset
		PF	66, F	Pfizer/BioNTech Second dose	28	New onset
		PV	73, F	Pfizer/BioNTech Third dose	28	New onset
		PV	63, F	AstraZeneca First dose	28	New onset
Alami et al. [43]	1	PF	44, M	Sinopharm First dose	7	New onset
Lansang et al. [44]	1	IgA pemphigus	64, M	Moderna Second dose	20	New onset
eWan et al. [45]	2	BP	50, F	Pfizer/BioNTech Second dose	14	New onset
		BP	82, M	Pfizer/BioNTech Second dose	3	New onset
Afacan et al. [46]	7	BP	Medium Age:75.2 F: 5 M: 2	CoronaVac:7 First dose: 1 Second dose: 4 Third dose: 2	Medium time: 18.7	New onset: 4 Recurrence: 3
Matsumoto et al. [47]	1	PF	73, M	Unspecified First dose	22	New onset
Rungraungrayabkul et al. [48]	1	Oral mucous membrane pemphigoid	74, F	Pfizer/BioNTech First dose	21	New onset
Pourani et al. [49]	1	PF	75, M	Sinopharm Third dose	14	New onset
Yıldırıcı et al. [50]	1	PF	65, M	Pfizer/BioNTech First dose	30	New onset
Bardazzi et al. [51]	4	BP	76, F	Pfizer/BioNTech Third dose	12	New onset
		BP	79, M	Pfizer/BioNTech Third dose	9	New onset
		BP	57, F	Moderna Third dose	7	Recurrence
		BP	62, M	Pfizer/BioNTech Third dose	7	Recurrence
Mustin et al. [52]	1	Pemphigoid Gestationis	36, F	Pfizer/BioNTech Second dose	10	New onset
Gui et al. [53]	2	Pemphigus vegetans	25, M	Pfizer/BioNTech Second dose	30	New onset
		PF	67, F	Moderna Second dose	14	New onset
Zou et al. [54]	3	PV	60, M	Moderna Second dose	7	New onset
		PV	40, F	Pfizer/BioNTech First dose	5	New onset
		PV	38, F	AstraZeneca First dose	7	New onset
Reis et al. [55]	1	PF	35, F	Pfizer/BioNTech Second dose	14	New onset
Akoglu [56]	3	PV	69, F	Unspecified Second dose	7	New onset
		PV	58, F	Unspecified Seconde dose	Unspecified	Recurrence
		PV	31, F	Pfizer/BioNTech Second dose	7	Recurrence
Khalayli et al. [57]	1	PV	50, F	Unspecified Second dose	7	New onset
Norimatsu et al. [58]	1	PV	86, M	Pfizer/BioNTech Second dose	1	New onset
Almasi-Nasrabadi et al. [59]	1	PF	62, F	AstraZeneca First dose	7	New onset
Falcinelli et al. [60]	1	Pemphigus erythematosus	63, F	Pfizer/BioNTech Second dose	2	New onset
Ong et al. [61]	1	PV	46, F	Moderna First dose	7	Recurrence
Saffarian et al. [62]	1	PV	76, F	Sinopharm Second dose	30	New onset
Gambichler et al. [63]	2	BP	80, M	Pfizer/BioNTech First dose	7	New onset
		BP	89, M	Pfizer/BioNTech First dose	2	New onset
Agharbi et al. [64]	1	PV	72, M	Pfizer/BioNTech Second dose	7	New onset

**Table 2:** Patient characteristics, clinical manifestations, timing to onset of symptoms, type and dose of COVID-19 vaccination received in patients with pemphigoid after vaccination. BP: bullous pemphigoid.

	Number of Patients (n = 83)
**Sex**	
Male	46 (55.4%)
Female	37 (44.6%)
**Mean Age (SD)**	72 ± 12.7
**Clinical Manifestations**	
BP	78 (94%)
Others	5 (6%)
**Days to symptom onset (SD)**	11 ± 10.8
**New onset diagnosis**	43 (51.8%)
**Recurrence**	19 (22.9%)
**Unspecified**	21 (25.3%)
**Type of COVID-19 vaccination**	
BNT162b2 (Pfizer-BioNTech)	49 (59%)
mRNA-1273 (Moderna)	11 (13.3%)
ChAdOx1 nCoV-19 AZD1222 (AstraZeneca)	11 (13.3%)
BBIBP-CorV (Sinopharm)	4 (4.8%)
CoronaVac (Sinovac)	7 (8.4%)
Unspecified	1 (1.2%)
**Vaccination dose**	
1st dose	34 (41%)
2nd dose	38 (45.8%)
3rd dose	8 (9.6%)
Unspecified	3 (3.6%)

**Table 3:** Patient characteristics, clinical manifestations, timing to onset of symptoms, type and dose of COVID-19 vaccination received in patients with pemphigus after vaccination. PV: pemphigus vulgaris (PV); PF: pemphigus foliaceus.

	Number of Patients (n = 59)
**Sex**	
Male	25 (42.4%)
Female	34 (57.6%)
**Mean Age (SD)**	55 ± 16.6
**Clinical Manifestations**	
PV	33 (55.9%)
PF	19 (32.2%)
Others	7 (11.9%)
**Days to symptom onset (SD)**	14 ± 11.7
**New onset diagnosis**	46 (78%)
**Recurrence**	13 (22%)
**Type of COVID-19 vaccination**	
BNT162b2 (Pfizer-BioNTech)	29 (49.2%)
mRNA-1273 (Moderna)	8 (13.5%)
ChAdOx1 nCoV-19 AZD1222 (AstraZeneca)	10 (16.9%)
ChAdOx1 nCoV-19 (Covishield)	1 (1.7%)
BIv1-CoViran	1 (1.7%)
BBIBP-CorV (Sinopharm)	6 (10.2%)
Unspecified	4 (6.8%)
**Vaccination dose**	
1st dose	20 (33.9%)
2nd dose	24 (40.7%)
3rd dose	9 (15.2%)
4th dose	1 (1.7%)
Unspecified	5 (8.5%)

## Data Availability

Data are reported in the current study.
